# Patient Comfort and Preference During Digital Versus Conventional Impressions for Fixed Prosthodontic Rehabilitation: A Comparative Study

**DOI:** 10.7759/cureus.116774

**Published:** 2026-09-23

**Authors:** Amit Sharma, Priyanka Batra, Avneet Kaur, Piyush Sharma, Aprajita Mittal, Ria Arora

**Affiliations:** 1 Department of Prosthodontics and Crown and Bridge, Luxmi Bai Institute of Dental Sciences and Hospital, Patiala, IND; 2 Department of Periodontology and Oral Implantology, Luxmi Bai Institute of Dental Sciences and Hospital, Patiala, IND

**Keywords:** dental impression technique, fixed partial denture, intraoral scanners, patient comfort, patient preference

## Abstract

Introduction: Digital impressions are increasingly being used in fixed prosthodontics; however, comparative data on patient-reported comfort and preferences in routine clinical settings remain limited. Thus, this study aimed to compare patient comfort, discomfort symptoms, preferences, and impression time between digital and conventional impression techniques for fixed prosthodontic rehabilitation.

Materials and methods: This was a prospective crossover clinical study of adult patients requiring single crowns or short-span fixed partial dentures. Each patient underwent both digital impression using an intraoral scanner and a conventional polyvinyl siloxane impression, with a 14-day interval between procedures. Patient-reported outcomes were assessed immediately after each procedure using a study-specific questionnaire with established face and content validity. Comfort and overall experience were assessed using a 0-10 visual analog scale (VAS), while gagging/choking, breathing difficulty, and anxiety/nervousness were assessed using ordinal response categories. Patient preference for future use of the same technique was recorded as a binary outcome. Impression time was also recorded. Paired *t*-tests were used for normally distributed continuous outcomes, and McNemar's test was used for paired binary outcomes. A two-sided *p* < 0.05 was considered statistically significant.

Results: A total of 30 patients were included in the study. Digital impressions produced significantly higher comfort scores (8.1 ± 1.2 vs. 5.8 ± 1.5; mean paired difference, 2.30; 95% CI, 1.76 - 2.84; p < 0.001) and overall experience scores (8.2 ± 1.1 vs. 6.0 ± 1.4; mean paired difference, 2.20; 95% CI, 1.70 - 2.70; p < 0.001) than conventional impressions. Gagging/choking was absent in 25 (83.3%) patients with digital impressions compared to 14 (46.7%) with conventional impressions (p = 0.004). Breathing difficulty was absent in 28 (93.3%) and 20 (66.7%) patients with digital impressions and conventional impressions, respectively (p = 0.013), and anxiety/nervousness was absent in 22 (73.3%) and 13 (43.3%) patients with digital impressions and conventional impressions, respectively (p = 0.016). A total of 22 patients (73.3%) preferred the digital technique for future treatment compared with six (20.0%) who preferred the conventional technique (p < 0.001). The mean impression time was significantly shorter with digital than with conventional impressions (5.1 ± 1.1 vs. 8.4 ± 1.7 minutes; mean paired difference, 3.30 minutes; 95% CI, 2.55 - 4.05; p < 0.001).

Conclusion: Digital impressions were associated with greater patient-reported comfort, fewer discomfort symptoms, greater preference, and shorter impression time than conventional polyvinyl siloxane impressions in patients who underwent fixed prosthodontic rehabilitation.

## Introduction

Digital dentistry has transformed prosthodontic practice by introducing intraoral scanners that replace traditional elastomeric impression materials [1]. Conventional impressions using polyvinyl siloxane (PVS) or similar materials are still widely used and clinically reliable. However, they frequently cause patient discomfort, such as gagging, unpleasant taste, breathing difficulty, and anxiety [2]. These problems can reduce patient acceptance and make the overall treatment experience unpleasant, especially for individuals with a sensitive gag reflex.

Intraoral scanners capture oral structures optically without trays or impression materials. This method is generally faster, cleaner, and more comfortable for patients [3]. Several studies have reported higher patient acceptance and lower procedural discomfort with digital impressions compared with conventional techniques [4]. Despite these advantages, many clinicians continue to rely on conventional methods, and comparative data focusing specifically on patient-reported comfort and preference during impression making remain limited in routine clinical settings. Patient-reported outcome measures are becoming increasingly important in modern dentistry. Treatment success is no longer judged only by clinical accuracy but also by how comfortable and acceptable the procedure feels to the patient [5]. Simple tools such as the visual analog scale (VAS) and short questionnaires can effectively capture subjective experiences without requiring complex instruments [6].

There is a need for straightforward clinical studies that evaluate patient comfort and preference between digital and conventional impressions using free and easy-to-administer questionnaires. Such evidence can help clinicians choose the more patient-friendly method in daily practice. This study aimed to compare patient-reported comfort, overall experience, discomfort symptoms, preference, and impression time between digital and conventional impression techniques used for fixed prosthodontic rehabilitation. The specific objectives were to compare comfort and overall experience using a 0-10 VAS, evaluate gagging/choking, breathing difficulty, and anxiety/nervousness associated with each technique, determine patients’ preference for each impression technique for future treatment, and compare the time required to complete each impression procedure. The a priori hypothesis was that digital impressions would result in higher patient-reported comfort than conventional impressions.

## Materials and methods

This was a comparative clinical study conducted at the Department of Prosthodontics and Crown and Bridge of Luxmi Bai Institute of Dental Sciences and Hospital, Patiala, Punjab, India, over a period of nine months from February 2024 to October 2024. Prior to the commencement of the study, ethical clearance was obtained from the Ethics Committee of Luxmi Bai Institute of Dental Sciences and Hospital (LBDH/26/46, dated January 15, 2024). Written informed consent was obtained from each participant after a detailed explanation of the study purpose, procedures, and their right to withdraw at any time without affecting their treatment. The study was conducted in accordance with the ethical principles outlined in the Declaration of Helsinki.

Both digital and conventional impression techniques are routinely performed in the department as part of the standard care for patients requiring fixed prosthodontic rehabilitation. The present study compared patient-reported comfort and preference between these two routinely used clinical procedures. No additional or experimental interventions were introduced beyond the normal treatment protocol of the department. A within-participant crossover design was selected because patient-reported comfort, discomfort, and preference are subjective outcomes that may vary substantially between individuals. Having each participant experience both impression techniques allowed a direct comparison of the two procedures while minimizing inter-individual variability. The additional impression procedure was limited to the research comparison, was performed using routine clinical techniques, and was considered to involve minimal additional risk or discomfort. Participants were informed of the study procedures and the need to undergo both impression techniques before providing written informed consent.

Sample size

The sample size was calculated using G*Power software version 3.1.9.7 (Heinrich-Heine-Universität Düsseldorf, Düsseldorf, Germany) for a paired t-test appropriate to the crossover design. Based on data from the randomized crossover study by Yilmaz et al. [7], a mean difference of 1.5 units on the VAS with a standard deviation of the differences of 1.8 was assumed. With 80% statistical power, a 5% alpha error, and a two-tailed test, a minimum of 14 participants was required. An anticipated dropout rate of 10% was considered during study planning; however, to provide a more conservative sample and account for potential incomplete responses or exclusions, the target sample was increased to 30 participants.

Eligibility criteria

Adult patients aged 18-70 years who required fixed prosthodontic rehabilitation in the form of single crowns or short-span fixed dental prostheses, had adequate oral hygiene, and were willing to participate were included in the study. The exclusion criteria were severe periodontal disease, active oral infection, uncontrolled systemic diseases, cognitive impairment that would prevent reliable questionnaire completion, a history of severe gag reflex that would make any impression procedure impossible, and previous prosthodontic treatment within the preceding six months.

Procedure

Each participant underwent both impression procedures, with the sequence of digital and conventional impressions randomized in a 1:1 ratio using a computer-generated randomization sequence. Participants were allocated to either the digital-first sequence (digital impression followed by conventional impression) or the conventional-first sequence (conventional impression followed by digital impression), with a 14-day interval between the two procedures to minimize potential carryover effects. A 14-day interval was selected between the two impression procedures to minimize potential carryover effects while allowing sufficient time for resolution of any transient discomfort associated with the first procedure.

All impressions (both digital and conventional) were performed by a single calibrated and experienced prosthodontist to eliminate inter-operator variability. The calibration of the operator was performed prior to the start of the study. The operator underwent a structured training session that included a theoretical review of both impression protocols, followed by a practical demonstration. The operator then performed 10 digital scans using the TRIOS intraoral scanner (3Shape, Copenhagen, Denmark) and 10 conventional putty-wash impressions on volunteer subjects who were not part of the main study. These trial impressions were evaluated independently by two senior prosthodontists for completeness, accuracy of margins, absence of voids or distortions, and adherence to manufacturer and standard protocols. Feedback was provided, and the process was repeated until the senior evaluators certified the operator’s consistency and proficiency. Only after successful calibration was the main study initiated. The same calibrated operator performed every impression throughout the study period under identical clinical conditions.

Digital Impressions

For the digital impression procedure, scanning was performed using a TRIOS intraoral scanner in accordance with the manufacturer’s recommended scanning protocol. Soft-tissue isolation and moisture control were maintained using cotton rolls and high-volume suction. The scanner tip was positioned and moved systematically over the prepared tooth or teeth, adjacent teeth, and relevant occlusal surfaces to obtain complete digital records. The scan was continuously evaluated on the chairside monitor, and additional scanning was performed when necessary to ensure complete capture of the preparation margins and surrounding structures. The final digital impression was accepted only when the operator confirmed adequate coverage and absence of clinically relevant gaps or missing areas.

Conventional Impressions

For the conventional impression procedure, impressions were obtained using Aquasil Ultra addition silicone (PVS) impression material (Dentsply Sirona Inc., Charlotte, North Carolina, United States) with standardized tray selection, following a two-step putty-wash technique. The tray was selected to provide adequate coverage of the dentition and sufficient space for the impression material. Tray adhesive was applied according to the manufacturer’s instructions. Soft-tissue management and moisture control were standardized and performed using cotton rolls and high-volume suction, consistent with the digital impression procedure. The putty material was mixed and loaded into the tray, and the preliminary impression was made and allowed to set according to the manufacturer’s recommended setting time. A light-body PVS wash material was subsequently applied to the prepared tooth or teeth, and the putty impression was reseated intraorally. After complete setting, the impression was carefully removed, inspected for completeness, marginal detail, voids, and other clinically relevant defects, and disinfected according to standard infection-control protocols. The final impression was accepted when adequate capture of the preparation margins and relevant surrounding structures was confirmed.

Study instrument

A structured, self-administered, study-specific questionnaire (see Appendices) was used to assess patient-reported outcomes associated with each impression procedure. The questionnaire comprised five items. The first two items assessed comfort during the impression procedure and overall experience using a 10-cm VAS. For each VAS, the left and right endpoints were anchored at 0 (“very uncomfortable”/“very bad”) and 10 (“very comfortable”/“excellent”), respectively. Patients were instructed to place a vertical mark on the scale corresponding to their response. The distance from the left endpoint to the patient’s mark was measured in centimeters using a ruler and recorded as a score from 0 to 10, with higher scores indicating greater comfort and a better overall experience. The third item assessed three procedure-related discomfort symptoms such as gagging/choking, breathing difficulty, and anxiety/nervousness, using a four-point ordinal scale: none = 0, mild = 1, moderate = 2, and severe = 3. The fourth item assessed whether the patient would prefer the same impression technique if the procedure were required again in the future, with responses recorded as yes or no. The fifth item provided an optional open-ended space for additional comments regarding the impression procedure.

The face and content validity of the questionnaire were evaluated by a panel of three senior prosthodontists with more than 10 years of clinical and academic experience. The experts independently rated each item for relevance, clarity, and completeness using a four-point scale (1 = not relevant, 2 = somewhat relevant, 3 = quite relevant, 4 = highly relevant). The content validity index (CVI) for individual items ranged from 0.89 to 1.00, and the overall scale CVI was 0.94, indicating excellent content validity [8]. Minor wording changes suggested by the experts were incorporated. Face validity was further confirmed by asking five patients (not part of the main study) whether the questions were easy to understand and relevant to their experience; all reported the questionnaire to be clear and appropriate.

Subsequently, a pilot study was conducted on 10 patients (five digital and five conventional sequences) who were not included in the final sample. The pilot confirmed that the questionnaire could be completed independently in two to three minutes, that the instructions were unambiguous, and that no additional items were required. Based on the pilot feedback, the layout was slightly adjusted for better readability, and the severity categories were clearly labelled. The final version of the questionnaire was therefore considered valid and suitable for use in the main study. Immediately after completing each impression procedure, the questionnaire was handed to the patient in a quiet area of the clinic. Patients completed the form themselves under the supervision of the investigator, who clarified any doubts without influencing their responses. Completed questionnaires were collected on the same day and checked for completeness. 

Outcomes

The primary outcome was patient-reported comfort measured using the 0-10 VAS. Secondary outcomes included overall experience, gagging/choking, breathing difficulty, anxiety/nervousness, patient preference, and impression time. Blinding of participants and the operator was not feasible because the digital and conventional impression procedures were visibly and procedurally different. However, patients completed the questionnaire immediately after each procedure without access to their previous responses, and the investigator collected and recorded the completed questionnaires without suggesting or influencing responses.

The time required to complete each impression procedure was recorded using a digital stopwatch. For the digital impression, timing commenced when intraoral scanning was initiated and ended when the complete digital scan was obtained and accepted by the operator as clinically adequate. For the conventional impression, timing commenced at the beginning of impression material manipulation and ended after removal of the set impression from the patient’s mouth and confirmation of its clinical adequacy. If additional scanning or impression procedures were required because of incomplete or defective capture, the additional time was included in the total procedure time. The same operator recorded the procedure time for both techniques using the same timing method throughout the study.

Data analysis

All data were entered into Microsoft Excel 2019 (Microsoft Corporation, Redmond, Washington, United States) and subsequently analyzed using IBM SPSS Statistics for Windows, version 26.0 (IBM Corp., Armonk, New York, United States). Continuous variables, including age, VAS scores, and impression time, were summarized as mean ± standard deviation (SD), whereas categorical variables, including sex, prosthesis type, symptom categories, and patient preference, were summarized as frequencies and percentages. The normality of the within-participant differences for continuous outcomes was assessed using the Shapiro-Wilk test. For normally distributed paired differences, comparisons between digital and conventional impressions were performed using paired t-tests. Discomfort symptoms were dichotomized as none versus any symptom (mild, moderate, or severe) and compared between the two paired impression techniques using McNemar’s test. Patient preference, recorded as a binary paired outcome, was also analyzed using McNemar’s test. All reported p-values were two-sided and unadjusted for multiple comparisons. The VAS-based comfort outcome was considered the primary outcome, while the remaining patient-reported and procedural outcomes were considered secondary outcomes. A significance level of p < 0.05 was used for statistical inference, and the secondary outcomes were interpreted with consideration of the multiple-comparison context.

## Results

A total of 30 participants completed both procedures, with no dropouts, and were included in the final analysis. The mean age of the participants was 36.8 ± 10.2 years. Of the 30 participants, 18 (60.0%) required single crowns and 12 (40.0%) required short-span fixed partial dentures (Table 1).

**Table 1:** Baseline demographic and clinical characteristics of the study participants (N = 30) Continuous data are presented as mean and standard deviation (SD), while categorical data are presented as frequency and percentage, FPD: fixed partial denture

Variable		Value
Age (years), mean ± SD		36.8 ± 10.2
Sex, n (%)	Male	16 (53.3%)
	Female	14 (46.7%)
Type of prosthesis –, n (%)	Single crown	18 (60.0%)
	Short-span FPD	12 (40.0%)

Patient comfort and overall experience were significantly better with the digital impression technique than with the conventional technique. The mean VAS score for comfort was 8.1 ± 1.2 for digital impressions and 5.8 ± 1.5 for conventional impressions (mean paired difference, 2.30; 95% CI, 1.76 - 2.84; p < 0.001). Similarly, the overall experience score was 8.2 ± 1.1 for digital impressions versus 6.0 ± 1.4 for conventional impressions (mean paired difference 2.20; 95% CI, 1.70 - 2.70; p < 0.001) (Table 2).

**Table 2:** Comparison of patient comfort and overall experience scores (VAS) between digital and conventional impressions (crossover analysis). NOTE: Intergroup comparison was done using paired t-test; p < 0.05: statistically significant

Parameter	Digital Impression, mean ± SD	Conventional impression, mean ± SD	Mean paired difference (95% CI)	t value	p-value
Comfort during impression (0–10)	8.1 ± 1.2	5.8 ± 1.5	2.30 (1.76 - 2.84)	8.71	< 0.001
Overall experience of impression (0–10)	8.2 ± 1.1	6.0 ± 1.4	2.20 (1.70 - 2.70)	8.04	< 0.001

Digital impressions were associated with significantly fewer discomfort symptoms than conventional impressions. Gagging/choking was absent in 25 patients (83.3%) during digital impressions compared with 14 patients (46.7%) during conventional impressions (McNemar χ² = 8.31, p = 0.004). Similarly, breathing difficulty was absent in 28 patients (93.3%) with digital impressions compared with 20 patients (66.7%) with conventional impressions (McNemar χ² = 6.13, p = 0.013). Anxiety/nervousness was absent in 22 patients (73.3%) during digital impressions compared with 13 patients (43.3%) during conventional impressions (McNemar χ² = 5.82, p = 0.016). Thus, digital impressions were associated with significantly lower patient-reported gagging, breathing difficulty, and anxiety than conventional impressions (Table 3).

**Table 3:** Comparison of discomfort symptoms between digital and conventional impression techniques. NOTE: Any symptom includes mild, moderate, or severe symptoms. Paired comparisons were performed using McNemar’s test after dichotomization into none versus any symptom; p < 0.05 was considered statistically significant.

Symptom		Digital impression, n (%)	Conventional impression, n (%)	McNemar χ²	p-value
Gagging/choking	None	25 (83.3)	14 (46.7)	8.31	0.004
	Any symptom	5 (16.7)	16 (53.3)		
Breathing difficulty	None	28 (93.3)	20 (66.7)	6.13	0.013
	Any symptom	2 (6.7)	10 (33.3)		
Anxiety/nervousness	None	22 (73.3)	13 (43.3)	5.82	0.016
	Any symptom	8 (26.7)	17 (56.7)		

Patient preference significantly favored the digital impression technique. A total of 22 patients (73.3%) preferred the digital impression method for future treatment, whereas six patients (20.0%) preferred the conventional impression method. The paired comparison demonstrated a statistically significant difference in preference between the two techniques (McNemar χ² = 14.22, p < 0.001) (Table 4).

**Table 4:** Patient preference for digital and conventional impression techniques for future treatment. Data are presented as frequency and percentage, The preference outcome was analyzed as a paired binary variable using McNemar’s test, p < 0.05 was considered statistically significant. McNemar χ² = 14.22; p < 0.001.

Preference after digital impression	Preference after conventional impression: Yes, n (%)	Preference after conventional impression: No, n (%)	Total, n (%)
Yes	5 (16.7)	17 (56.7)	22 (73.3)
No	1 (3.3)	7 (23.3)	8 (26.7)
Total	6 (20.0)	24 (80.0)	30 (100.0)

The impression time was significantly shorter with the digital technique (5.1 ± 1.1 minutes) than with the conventional technique (8.4 ± 1.7 minutes), with a mean paired difference of 3.30 minutes (95% CI, 2.55 - 4.05; p < 0.001) (Table 5).

**Table 5:** Comparison of impression time between digital and conventional techniques. NOTE: Intergroup comparison was done using paired t-test, p < 0.05: statistically significant, CI: confidence interval.

Parameter	Digital impression, mean ± SD	Conventional impression, mean ± SD	Mean difference (95% CI)	t value	p-value*
Impression time (minutes)	5.1 ± 1.1	8.4 ± 1.7	3.30 (2.55 - 4.05)	11.78	< 0.001

## Discussion

This study demonstrated that digital impressions using an intraoral scanner were significantly more comfortable and better accepted by patients than conventional polyvinyl siloxane impressions for fixed prosthodontic rehabilitation. The mean VAS comfort and overall experience scores were markedly higher for the digital technique than for the conventional technique. These findings are consistent with previous crossover trials. Yilmaz et al. reported significantly higher comfort scores for digital scans compared with conventional impressions and noted that 84.6% of patients considered the digital method more comfortable [7]. Joda and Brägger similarly found higher convenience scores for intraoral scanning (78.6 versus 53.6 on a 0-100 scale) and reported that all patients preferred the digital workflow for future treatment [9].

The lower frequency of gagging, breathing difficulty, and anxiety observed with digital impressions in the present study also aligns with the broader literature. Systematic reviews and meta-analyses have confirmed that intraoral scanning reduces discomfort, nausea, unpleasant taste, and breathing difficulty compared with conventional impression making [10,11]. The absence of impression trays and elastomeric materials eliminates the main triggers of the gag reflex and the sensation of airway obstruction, which explains the substantially higher proportion of patients reporting “none” for these symptoms during digital scanning.

Patient preference strongly favored the digital method. This preference rate is comparable to the figures reported in earlier studies, which have consistently shown that a clear majority of patients would choose digital impressions if given the option again [2,12]. The shorter chairside time recorded for digital impressions further supports patient acceptance and is in agreement with several previous investigations that demonstrated greater time efficiency with intraoral scanners once the operator is experienced [13-15].

Although the present study found that digital impressions were significantly faster than conventional polyvinyl siloxane impressions, a network meta-analysis by Sivaramakrishnan et al. reported an overall longer operating time for digital techniques [11]. This discrepancy is most likely related to differences in scanner systems, operator experience, and the extent of the scan (full-arch versus single-tooth or short-span preparations). In the present study, all scans were performed by a single calibrated prosthodontist using the TRIOS scanner for limited preparations, which may explain the observed time advantage of the TRIOS scanner. However, patient preference remained consistently in favor of digital impressions in both the present study and the meta-analysis.

The use of the TRIOS intraoral scanner in the present study is likely to have contributed to favorable patient-reported outcomes and shorter chairside time. The TRIOS system features continuous video-based scanning, real-time feedback, and a relatively small scanner tip, which facilitates the efficient capture of prepared teeth and adjacent structures with minimal patient discomfort [16]. Previous clinical studies that employed the TRIOS scanner similarly reported high patient preference and reduced procedural time compared with conventional impressions, supporting the findings of the current investigation [16,17].

Clinical implications

The results reinforce the clinical value of adopting intraoral scanning as the primary impression technique for single crowns and short-span fixed partial dentures whenever possible. Improved patient comfort can enhance treatment acceptance, reduce appointment stress, and potentially improve cooperation, particularly in patients with a sensitive gag reflex or elevated dental anxiety. The time saving observed may also increase clinical efficiency and allow more appointments to be completed within the same working day.

Limitations

This study has several limitations. The sample size, although adequately powered for the primary VAS outcomes, remains relatively modest and was drawn from a single center, which may limit generalizability. Blinding of participants and the operator was not feasible because the two impression techniques were readily distinguishable, and potential expectation bias cannot therefore be excluded. Formal testing for carryover and period effects was not performed, although randomization of treatment sequence and a 14-day interval were used to minimize potential sequence effects. Multiple secondary comparisons were performed without adjustment for multiplicity and should therefore be interpreted cautiously. Only one intraoral scanner (TRIOS) and one conventional material (PVS) were evaluated; results may differ with other systems or materials. Operator experience with digital scanning can influence both comfort and time outcomes, and all impressions were performed by a single calibrated prosthodontist. No participants were lost to follow-up, as all 30 participants completed both procedures. Finally, patients with a severe gag reflex that would have prevented completion of either impression procedure were excluded, which limits generalizability to patients with pronounced gag sensitivity. Formal testing for carryover or period effects between sequence groups was not performed. In addition, inter-rater reliability for VAS ruler measurements was not assessed, as the VAS marks were measured directly on a standardized 10-cm scale. These factors should be considered when interpreting the reproducibility of the findings. Finally, long-term clinical outcomes of the resulting restorations were not assessed. Larger multicenter trials incorporating different scanners and longer follow-up would strengthen the evidence base.

## Conclusions

Digital impressions demonstrated significantly greater patient comfort, lower frequencies of gagging, breathing difficulty, and anxiety, greater preference for future use, and shorter chairside time compared with conventional polyvinyl siloxane impressions. These findings indicate potential patient-experience and time-efficiency advantages of digital impressions for single crowns and short-span fixed partial dentures within the studied population; however, their generalizability to broader routine prosthodontic practice may be limited by the single-center, single-scanner, and single-operator design.

## Appendices

Study questionnaire 

**Table 6:** Patient questionnaire used in the study. VAS scores are recorded on a 0–10 scale by measuring the distance from the left endpoint in centimetres. Symptom severity is scored as none = 0, mild = 1, moderate = 2, and severe = 3. For statistical analysis, symptom severity is dichotomized as none versus any symptom. Preference is recorded as a binary outcome (yes/no). VAS: visual analogue scale; FPD: fixed partial denture; PVS: polyvinyl siloxane

Section	Item	Response
Patient details	Patient ID	
	Date	
	Age (years)	
	Sex	□ Male □ Female
	Type of prosthesis	□ Single crown □ Short-span FPD
	Impression technique used today	□ Digital (TRIOS scanner) □ Conventional (PVS)
	Procedure period	□ First impression □ Second impression (after 14-day interval)
Instructions	Please answer the following questions based on the impression procedure you have just undergone. Mark the point on each scale that best represents your experience. There are no right or wrong answers.	
Section A: Comfort and experience	1. Comfort during the impression procedure	0 (Very uncomfortable) ─────────────── 10 (Very comfortable)
	Score	______ / 10
	2. Overall experience of the impression procedure	0 (Very bad) ───────────────────────── 10 (Excellent)
	Score	______ / 10
Section B: Discomfort symptoms	3. Did you experience any of the following during the impression? Tick one box for each symptom.	
	Gagging/choking	□ None □ Mild □ Moderate □ Severe
	Breathing difficulty	□ None □ Mild □ Moderate □ Severe
	Anxiety/nervousness	□ None □ Mild □ Moderate □ Severe
Section C: Preference	4. Would you prefer the same impression method again if needed in the future?	□ Yes □ No
	5. Any other comments about the impression procedure? (Optional)	
